# Antioxidant Marine Hydrolysates Isolated from Tuna Mixed Byproducts: An Example of Fishery Side Streams Upcycling

**DOI:** 10.3390/antiox13081011

**Published:** 2024-08-19

**Authors:** Federica Grasso, María Mercedes Alonso Martínez, Federica Turrini, Diego Méndez Paz, Rebeca Vázquez Sobrado, Valentina Orlandi, Marte Jenssen, Kjersti Lian, Junio Rombi, Micaela Tiso, Elisabetta Razzuoli, Celina Costas, Raffaella Boggia

**Affiliations:** 1 Department of Pharmacy, University of Genova, Viale Cembrano 4, 16148 Genova, Italy; federica.grasso@edu.unige.it (F.G.); valentina.orlandi@edu.unige.it (V.O.); raffaella.boggia@unige.it (R.B.); 2 ANFACO-CECOPESCA, Department of Circular Economy, Colexio Universitario, 36310 Vigo, Spain; malonso@anfaco.es (M.M.A.M.); dmendez@anfaco.es (D.M.P.); rebeca.vazquez@anfaco.es (R.V.S.); ccostas@anfaco.es (C.C.); 3 National Center for the Development of New Technologies in Agriculture (Agritech), 80121 Napoli, Italy; 4 Nofima, Muninbakken, 9-13, 9019 Tromsø, Norway; marte.jenssen@nofima.no (M.J.); kjersti.lian@nofima.no (K.L.); 5 MICAMO LAB, Via XX Settembre 33/10, 16121 Genova, Italy; j.rombi@micamo.com (J.R.); m.tiso@micamo.com (M.T.); 6 Sezione di Genova Portualità Marittima, Istituto Zooprofilattico Sperimentale del Piemonte, Liguria e Valle d’Aosta, 16129 Genova, Italy; elisabetta.razzuoli@izsto.it; 7 National Biodiversity Future Center (NBFC), 90133 Palermo, Italy

**Keywords:** tuna mixed biomass, enzymatic hydrolysis, extensively hydrolyzed peptides, antioxidant activity, anti-inflammatory capacity, anti-osteoporotic capacity

## Abstract

The aim of this research is to propose simple and scalable processes to obtain bioactive peptides extensively hydrolyzed starting from a tuna mixed biomass. The upcycling of this powdered biomass is challenging since it comes from the unsorted industrial side streams of the tuna canning process (cooked residues from fillet trimming) after a patented mild dehydration useful for preventing its degradation until its exploitation. Two different protocols were proposed, with and without the inclusion of an exogenous enzyme (Enzymatic-Assisted Extraction, EAE), with no relevant differences in yields (24% vs. 22%) and a comparable amino acid composition. Nevertheless, the former protocol (with EAE) provided peptides with an average molecular weight of 1.3 kDa, and the second one (without EAE) provided peptides with an average molecular weight of 2.2 kDa. The two corresponding types of tuna protein hydrolysates (Enzymatic Hydrolysates (EH) and Non-Enzymatic Hydrolysates (NEH)) were characterized by proximate compositions, pH, color profile, amino acid analysis, FTIR spectra, and molecular weight distribution. In addition, several biological analyses were performed to assess their potential use as nutraceutical supplements: special attention has been paid to antioxidant activity using three different methods to quantify it. EH showed the most promising antioxidant activity which could be exploited also in other fields (e.g., biomaterials, cosmetics).

## 1. Introduction

According to the Food and Agriculture Organization (FAO), 165 million tons of aquatic animal foods were consumed globally in 2022, growing since 1961 at almost twice the annual rate of the world population. The apparent annual per capita consumption of aquatic animals went from 9.1 kg in 1961 to an estimated 20.7 kg in 2022 [1].

Fish is considered one of the foods with the highest nutritional value because of its vast array of health-promoting nutrients. Numerous studies have shown that dietary supplements containing fish nutrients are beneficial for treating a variety of inflammatory-related illnesses, including hyperlipidemia, ulcerative colitis, and cardiovascular diseases [2,3,4].

Nevertheless, the processing of seafood biomass creates large amounts of side streams in the form of solid products, including canning and fish meal, and effluents, such as cooking juice (broths), stick water, and washing water, which contains soluble proteins, that can be collected, concentrated, and utilized as a raw material to make protein hydrolysates [5]. In the latest reports, it is estimated that up to 70% of all tuna is discarded during tuna processing, producing about 22 million tonnes/year [6].

During the tuna canning process, besides the leftovers like heads, tails, fins, guts, and skin, another type of residue is produced after cooking, and it is represented by the discarded meat and pieces that are too big or too small or deformed to be canned. This side stream, which represents 5–10% of the initial tuna weight, is still a valuable source of nutrients whose final usage is often limited to animal feeding. Different processing factors, such as increasing temperature by cooking, can largely decrease the solubility of the proteins, mainly caused by denaturation [7]. Nevertheless, the high content of valuable nutrients deserves an upcycling process and not only a recycling process, as extracting high-added-value compounds that could be valorized in many sectors (e.g., hydrolyzed peptides).

Typically, in fishery side stream upcycling, it is standard practice to begin with carefully sorted biomass to minimize extraction efforts and enhance yields. Depending on the type of marine source, specific parts or tissues are selected and utilized based on their protein content and the feasibility of extracting them. As reported in the literature, starting with unsorted biomass is far more complex [8]. While this approach can be challenging, it sometimes offers an alternative that is worth exploring. Furthermore, when processing fish side streams to produce fish protein hydrolysates, the quality of the raw materials is crucial. As a result, managing fresh fish and side streams properly after harvest is essential to prevent denaturation and deterioration of myofibrillar proteins [9]. Although freezing is the most common industrial method for preserving biomass, it is highly expensive, both in terms of energy and money. On the contrary, dehydration represents a lower-cost alternative and makes biomass more easily manageable by reducing volume and weight. However, the main limitation of dehydration is the risk of oxidative degradation of this perishable biomass, so only mild and controlled dehydration could be considered a viable alternative [10].

Fishery side streams have gained a lot of attention from researchers since they can be converted via enzymatic hydrolysis into fish protein hydrolysates with high protein content and a balanced amino acid composition, as well as bioactive peptides that exhibit a wide range of potential bioactivities, including antioxidant, angiotensin-converting enzyme (ACE)-inhibiting, calcium-binding, and anticoagulant properties [11,12]. This represents a “recycling strategy” and an “upcycling strategy” that transforms low-value biological side streams, generally intended for animal feed, into new products or ingredients of higher value or utility.

Recently, peptides identified from different marine biological resources have shown great potential for treating a variety of diseases [13,14]. In case of tuna, different bioactive peptides were prepared from byproducts, such as dark muscle, milt, bone/frame, scale, roe, head, and viscera, and showed significant potential in the area of functional products. For example, different peptides were reported which displayed significant blood pressure lowering activity by restraining angiotensin-I converting enzyme (ACE) activity [15,16,17,18]. Other peptides presented remarkable radical-scavenging activity, lipid oxidation inhibition capability, and reducing power [19,20,21]. It is of relevance that in some cases its efficacy has been demonstrated in in vivo trials; Maneesai et al. [22] investigated whether supplementation with tuna protein hydrolysate (TPH) could alleviate cardiovascular complications induced by a high-fat diet (HFD) in rats. Wang et al. [23] illustrated that enzymatically hydrolyzed peptides from Skipjack (Katsuwonus pelamis) had a good regulating effect on inflammation and intestinal flora of UC model mice induced by DSS. Instead, Xiang et al. [24] reported the protective effect of tuna bioactive peptides in induced colitis mice. On the basis of their properties, peptides can thus be used in the development of functional foods and nutraceuticals.

Chemical and biological hydrolysis are the most often employed approaches for producing protein hydrolysates. The chemical technique exploits the hydrolysis reaction due to the presence of acids or bases. However, chemical hydrolysis is challenging to manage because of the harsh reaction and non-specific peptide bond cleaving [25]. On the contrary, biological hydrolysis such as enzymatic hydrolysis (EAE), with the addition of exogenous enzymes, and autolysis, i.e., the process carried out by endogenous proteolytic enzymes, are considered mild processes that involve the specificity of the enzyme employed [26].

This work, funded by the EcoeFISHent project [27], aims to meet the objectives of the 2030 Agenda for Sustainable Development and the ones that include protecting water resources as well as promoting responsible production and consumption. The purpose and the novelty of this work were specifically the upcycling of dehydrated cooked side streams coming from the tuna canning industry Generale Conserve (ASdoMAR^®^). This study presents two different approaches for extracting protein hydrolysates from a mixed fish powder obtained using an innovative mild dehydration technique and characterization of them while studying some of their potential biological properties.

## 2. Materials and Methods

### 2.1. Chemicals

The following chemicals and reagents were of analytical quality and provided by Sigma-Aldrich Chemical Company (Steinheim, Germany): Fluorescein, Phosphate Buffer Solution (PBS), Fetal Bovine Serum (FBS), antibiotic solution, 2′,7′-dichlorofluorescein diacetate, Hanks’ Balanced Salt Solution (HBSS), 4-(2-Hydroxyethyl)piperazine-1-ethanesulfonic acid (HEPES), Lipopolysaccharide (LPS), Hydrocortisone, 3-(4,5-dimethylthiazol-2-yl)-2,5-diphenyltetrazolium bromide, dimethyl sulfoxide, N-[3-(2-Furyl)acryloyl]-Phe-Gly-Gly, β-glycerophosphate, ascorbic acid, dexamethasone, bovine serum albumin (BSA), captopril, sulfuric acid, sodium hydroxide, hydrochloric acid, thiobarbituric acid, 1,1,3,3-tetramethoxypropane, trichloroacetic acid, 1-butanol, and isopropanol. The following chemicals and reagents were of analytical quality and provided by Thermo Scientific Group (Waltham, MA, USA): (2-2′-Azino-di-[3-ethylbenzthiazoline sulfonate], Trolox, 2,2,-azobis(2-methylpropionamidine) dihydrochloride). Büchi Labortechnik AG (Flawil, Switzerland) supplied 2% boric acid with Sher indicator and catalyst tablets for the Kjeldahl analysis. All the chemicals and reagents of HPLC-level were purchased from Waters (Milford, MA, USA). Ultrapure Milli-Q water (18 MΩ) was produced by a Millipore Milli-Q system (Bedford, MA, USA). The proteolytic enzyme, a serine hydrolase called 3G PBN-66 L, derived from the fermentation of *Bacillus licheniformis*, was provided by 3G SINCE 2000 (Barcelona, Spain). As for the Ferric Reducing Antioxidant Power (FRAP) assay, the kit (ab234626) was acquired from Abcam© (Abcam, Caliph, ML, Cambridge, UK).

### 2.2. Samples

**Figure 1:**
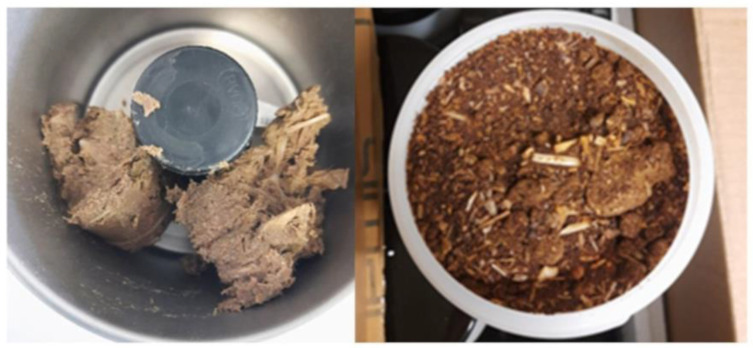
Cooked tuna side streams before (TFB) and after (TFP) the mild dehydration process.

The initial raw material was composed of mixed cooked tuna (Yellowfin Tuna) leftovers coming from the company Generale Conserve, ASdoMAR^®^ (Olbia, Italy), which produces canned tuna for human consumption. After a patented process of mild dehydration performed by Themis S.p.A. (Legnano, Italy) [28] the tuna fish biomass (TFB) was transformed into a heterogeneous powder, referred as tuna fish powder (TFP) (Figure 1), which was the starting point for the extraction of fish protein hydrolysates, both with and without the addition of an Alcalase to carry out an enzymatic hydrolysis extraction.

### 2.3. Microbiological Analysis

The microbiological investigation evaluated the effectiveness of the industrial dehydration procedure, currently under patent [28], in stabilizing and preserving this high perishable fish biomass. In particular, the ISO 15213-1:2023 official technique [29] was used to determine the sulfite-reducing Clostridia content. The Total Viable Count (TVC) was calculated using the ISO 4833-1:2013/Amd-1:2022 technique [30]. Microbiological characterization was performed on the following microorganisms: Coliforms (ISO 4832:2006) [31], *Salmonella* spp. (ISO 6579-1:2017/Amd 1:2020) [32], *Escherichia coli* β-gluc. +(ISO 16649-2:2001) [33], *Enterobacteriaceae* (ISO 21528-2:2017) [34], coagulase-positive *Staphylococci* at 37 °C (ISO 6888-2:2021) [35], and *Listeria monocytogenes* (ISO 11290-1:2017) [36]. Histamine has been assessed following Malle et al.’s procedure [37].

### 2.4. Lipid Oxidation by Peroxide Value and Thiobarbituric Acid Reactive Substances Assays

On this highly unsaturated organic biomass, and therefore easily subject to lipid oxidation, oxidation state monitoring was carried out to evaluate the impact of the initial dehydration process. To assess the level of lipid oxidation, Peroxide Value (PV), for the primary oxidation products, and a Thiobarbituric Acid Reactive Substances (TBARS) assay, for the secondary oxidation, were conducted directly on the starting materials, both pre- and post-dehydration treatment following the official method ISO 5.4.205 rev.1 2020, described by Hu et al. [38] with minor differences as reported below. The TBARS solution was obtained by dissolving 15 g of trichloroacetic acid and 0.75 g of thiobarbituric acid in a 100 mL solution containing 20 mL 0.5 M HCl, 40 mL isopropanol, and 40 mL 1-butanol. After weighing the samples in Pyrex tubes and adding the TBARS solution, the samples were incubated for 2 h at 95 °C in a water thermostatic bath (E200, LAUDA, Germany). After quickly cooling the tubes under tap water, the samples were measured at 532 nm using a UV–visible spectrophotometer (Agilent Cary 100 Varian Co., Santa Clara, CA, USA), and the calculations were made considering a calibration curve obtained with TMP (1,1,3,3-tetramethoxypropane) as the standard, in a range of 0.61–6.10 μM.

### 2.5. Extraction Process of Tuna Protein Hydrolysates with and without Adding Enzymes

The extraction of low-molecular-weight peptides (EH, Enzymatic Hydrolysates) from TFP was performed following the protocol proposed in a previous paper by Grasso et al. [10] but with some relevant modifications. Deionized water at a ratio of 1:6 (*w*/*v*) was included to a total of 1.35 kg of TFP, and the pH was checked to ensure that it was near the range of activity of the enzyme. The enzyme used for this extraction works efficiently in a range of pH between 6 and 10, reaching an optimum at pH 9.0. However, to avoid an increase in salt content and since the pH of the hydrolysate was considered suitable for the scope, no alkali solvent was added during the process. The enzyme Alcalase 3G PBN-66L (3G SINCE 2000, Barcelona, Spain) was included at 1%, and the reaction was carried out at 60 °C for 1 h in an incubator shaker (SKI 8R, Argo Lab, Kunshan, China). After the inactivation of the enzyme (80 °C, 5 min), the suspension was immediately filtered, passing first through a sieve of 200 µm, and then, the liquid was filtered under a vacuum with filter paper. The hydrolysate was stored at −32 °C until spray-drying using a B290 mini spray-dryer (Büchi Labortechnik AG, Flawil, Switzerland).

To obtain peptides with higher molecular weight (Non-Enzymatic Hydrolysates, NEH), the same protocol was followed, but the enzyme was omitted.

### 2.6. Proximate Analysis

After blending the TFP samples to obtain a more homogeneous powder, they were examined twice using official methods to determine their residual moisture (AOAC 950.46B), protein (AOAC 981.10), ashes (AOAC 942.05) [39], and lipid [40] contents. The same protocols were adopted to determine the proximate analysis of the extracted tuna protein hydrolysates (EH and NEH).

### 2.7. pH

Before the extraction of the tuna protein hydrolysates (EH and NEH), TFP was diluted in water in a 1:6 (*w*/*v*) ratio, and the pH was measured at room temperature (25 ± 1 °C) using a pH meter (HI5221, Hanna Instruments, Villafranca Padovana, Padova, Italy). As for the EH and NEH, the British Standard Institution’s 1975 procedure was followed with slight modifications, dissolving them in water at room temperature to create a 1% solution (g/mL).

### 2.8. Color Analysis: CIELab Color Space

To analyze the color of the EH and NEH, a double-beam UV–visible spectrophotometer (Agilent Cary 100 Varian Co., Santa Clara, CA, USA) equipped with an integrating sphere (Varian DRA) was used in the 300–900 nm range. The integrating sphere uniformly disperses light across all interior surfaces at a resolution of 1 nm. Triplicate analyses were performed considering a white Spectralon^®^ disk as a reference. Using the program Cary100 Color, the CIE D65 illuminant was used to automatically extract the CIELab coordinates L* (lightness), a* (greenish–reddish), and b* (bluish–yellowish) from the raw spectral data.

### 2.9. Amino Acid Profile

To assess the amino acid profiles of EH and NEH, an HPLC Acquity Arc (Waters Corp, Milford, MA, USA) integrated with a fluorescence detector, Waters 2475 and a Waters AccQ-Tag Amino Acids C18 Column, with a particle size of 3 µm (3.9 mm × 150 mm), was used. Following the procedure described by Lorenzo et al. [41], the hydrolysis was carried out starting from 100 mg of sample and 5 mL of 6 N hydrochloric acid at 110 °C for 24 h; then, 200 mL of distilled water was added to the hydrolyzed solution. The mixture was then filtered through a 0.45 μm filter. As for the derivatization, considering the protocol proposed by Domínguez et al. [42], 10 μL of the abovementioned solution was buffered with AccQ-Fluor borate buffer to pH 8.8 to obtain a total volume of 100 μL, and 20 μL of AcQ-Fluor reagent (3 mg/mL in acetonitrile) was added to initiate the reaction. The injection volume was 10 µL for each sample with a 1.0 mL/min flow rate in a working condition of 37 °C. As recommended by van Wandelen [43], the mobile phases were made starting with Waters AccQ-Tag Eluent A, acetonitrile, and Milli-Q ultrapure water. The detection was accomplished using a fluorescence detector with an excitation length of 250 nm and an emission length of 395 nm. The analyses were carried out in duplicate.

### 2.10. Molecular Structure by Attenuated Total Reflectance–Fourier-Transform Infrared Spectroscopy

The molecular structures of EH and NEH were evaluated employing Attenuated Total Reflectance–Fourier-Transform Infrared (ATR-FTIR) spectroscopy after recording a preliminary background. Using an FT-IR spectrophotometer (Perkin Elmer, Inc., Waltham, MA, USA), spectra were recorded at room temperature from 4000 to 600 cm^−1^ at a resolution of 4 cm^−1^, accumulating 8 scans per sample.

### 2.11. Average Molecular Weight by Size Exclusion Chromatography

Size Exclusion Chromatography (SEC) was used to determine the EH and NEH’ average molecular weights and molecular weight distributions following the procedure suggested by Wubshet et al. [44] with a few modifications. Sample solutions of 20 mg/mL were prepared for the analysis. A Prominence Shimadzu HPLC system (Shimadzu, Nishinokyo Kuwabara-cho, Nakagyo-ku, Japan) connected to a Prominence photodiode array detector (PDA) (Shimadzu) was used to carry out the analysis using a BioSep-SEC-s2000 column (300 × 7.8 mm) (Phenomenex, Værløse, Denmark). The wavelength of the PDA detection was set to 214 nm, with 640 ms intervals. The analyses were carried out in duplicate, injecting 10 μL each time and with chromatographic separation at 30 °C. Using 30% acetonitrile with 0.05% trifluoroacetic acid as the mobile phase, isocratic elution was performed for 17 min at a flow rate of 0.9 mL/min. Then, the column was washed with 0.10 M NaH_2_PO_4_ for three minutes and re-calibrated for 25 min using the previous mix of mobile phase. LabSolutionsTM CL Realtime Analysis software (Shimadzu) version 5.86 was used to monitor the chromatographic runs. The molecular weights were determined throughout the hydrolysates’ elution interval, which lasted between 5 and 17 min. The parallels were evaluated using Pearson correlations, based on retention time and intensity, and estimated by the PDA at 214 nm. A calibration curve made with molecular weight standards between 0.204 kDa and 30 kDa (Carbonic anhydrase, Lysozyme, Cytochrome c from bovine heart, Aprotinin from bovine lung, Insulin chain B oxidized from bovine pancreas, Renin substrate tridecapeptide porcine, Angiotensin II human, Bradykinin fragment 1–7, [D-Ala2]-Leucine encephalin, Val–Tyr–Val, and L-tryptophane) was used to calculate the molecular weights in Microsoft Excel version 2402 (Microsoft Corporation, Seattle, WA, USA).

### 2.12. Determination of Antioxidant Activity

#### 2.12.1. Ferric Reducing Antioxidant Power Assay

A commercial assay kit for FRAP was used to measure the total antioxidant activity [45] of EH and NEH. The antioxidant power is evaluated by the reaction involving the reduction of ferric iron (Fe III) to ferrous iron (Fe II), which produces a blue color with a maximum absorption value at 594 nm. The antioxidant capacity of the sample solutions was calculated using the ferrous ammonium sulfate standard curve, which had an R^2^ value of 0.998. The sample solutions were prepared in water at concentrations ranging from 25 to 200 mg/mL, and the results were reported as Fe II equivalents (mM).

#### 2.12.2. Trolox Equivalent Antioxidant Capacity Assay

The Trolox Equivalent Antioxidant Capacity TEAC radical cation decolorization assay was performed following the procedure of Re et al. [46] with some modifications. The radical is formed by the redox reaction between 7.5 mM ABTS and 2.47 mM potassium peroxydisulfate (Scharlab S.L. Barcelona, Spain), a strong oxidant. The resulting solution was kept in the darkness at room temperature for 12–16 h. For the assay, the ABTS^•+^ (2-2′-Azino-di-[3-ethylbenzthiazoline sulfonate]) radical solution was diluted to a final concentration of 0.15 mM and added to the sample or standard. Absorbances were measured in the darkness using a spectrophotometer (Spark, Tecan), at 734 nm wavelength for 5 min, recording measurements every minute. The absorbance decrease is produced in the presence of an antioxidant. The ABTS radical-scavenging capacity was determined using a standard curve of Trolox (4.7–300 µM). The results were expressed as µmol Trolox equivalents/g dry hydrolysate.

#### 2.12.3. Oxygen Radical Absorbance Capacity

The Oxygen Radical Absorbance Capacity (ORAC) assay was monitored following a modified method reported by López Méndez [47], Prior et al. [48], and Ou et al. [49]. It is a highly sensitive method which relies on the measurement of the fluorescence decay caused by the peroxyl free radicals that are in situ generated via thermal decomposition of an azo-compound AAPH (2,2,-azobis(2-methylpropionamidine) dihydrochloride)). The presence of an antioxidant causes the delay of the fluorescence quenching. The reaction is carried out via interaction of the sample or standard with 0.011 nM fluorescein and 12 mM AAPH (final concentration in well). Fluorescence data were recorded using a spectrophotometer (SpectraMax M5), with excitation and emission wavelengths of 485 and 520 nm, respectively. The fluorescence spectrum was registered every 2 min over 90 min. The ORAC capacity was determined using a standard curve of Trolox (13.2–150 µM). The results were calculated based on the differences in areas under the curves of fluorescence decay of the fluorescein between the blank and the sample (net area under the curve). The results were expressed as µmol Trolox equivalents/g dry hydrolysate.

#### 2.12.4. Cellular Antioxidant Activity Assay

The Cellular Antioxidant Activity (CAA) assay is performed using the human hepatoma cell line Hep G2 as described by Wolfe et al. [50] with some modifications. Briefly, HepG2 cells were grown in Eagle’s Minimum Essential Medium (EMEM) (ATCC-30-2003) supplemented with 10% FBS and antibiotic (solution of 10,000 units of penicillin and 10 mg streptomycin/mL) at 1%. The cultures were maintained in a 37 °C incubator with 5% CO_2_ and a humid atmosphere. During the logarithmic growth phase, cells (6 × 10^5^/mL) were seeded into a 96-well microplate (100 μL/well) and incubated for 24 h in a fully humidified atmosphere in a 5% CO_2_, 37 °C incubator. The cells were washed with 200 μL of PBS and incubated for an additional 2.5 h with a medium that contained diosmetin and 2′,7′-dichlorofluorescein diacetate (DCFH-DA) (final concentration of 25 μM) and different concentrations of each hydrolysate sample solution. Next, the cells were washed with 200 μL of PBS, after which 100 μL of a new medium made of HBSS plus 10 mM of HEPES containing 500 μM AAPH was added to each well. The 96-well microplate was placed in a spectrophotometer (SpectraMax M5, Molecular Devices LLC, San Jose, CA, USA) thermostated at 37 °C, and the fluorescence was monitored every 5 min for 1 h (λ ex/em, 485/520 nm). Each plate included triplicate control and blank wells. Control wells contained cells treated with DCFH-DA and oxidant; blank wells contained cells treated with dye and HBSS without oxidant. The fluorescence value was calculated using the method of Liao et al. [51]. All the results were presented as % of inhibition of oxidation (mean ± standard error).

### 2.13. Other Biological Tests

#### 2.13.1. Viability Assay

This test is carried out as a prior step to all biological tests to select the maximum sample concentrations that can be used without affecting cell viability, ensuring the reliability of the results obtained in each of the cell lines. Viability was assessed by the well-known MTT test, based on NAD(P)H-dependent cellular oxidoreductase enzymes that can reduce the tetrazolium dye, MTT (3-(4,5-dimethylthiazol-2-yl)-2,5-diphenyltetrazolium bromide), to insoluble formazan. A dimethyl sulfoxide (DMSO) solution is added to dissolve formazan product into a colored solution. The test is performed in 96-well plates, with a volume of 100 µL per well and with different concentrations depending on the cell line: 5 × 10^5^ cells/mL in RAW 264.7 (ATCC TIB-71) and 5 × 10^3^ cells/mL in HepG2 (ATCC^®^ HB-8065™). In practice, after treatment with the test compound for 24 h, 10 μL of 5 mg/mL MTT in PBS was added to each well before the plate was incubated in 5% CO_2_ at 37 °C for 4 h. Next, the solutions in the well were carefully removed, and 100 μL of DMSO was added to each well and incubated for 20 min. The absorbance of each well was subsequently measured at 570 nm of wavelength by a spectrophotometer (Molecular Devices LLC, USA). Cell viability was expressed as % of viability with respect to the control.

#### 2.13.2. Determination of ACE-Inhibitory Activity (Antihypertensive Capacity)

The ACE-inhibitory assay was measured spectrophotometrically using N-[3-(2-Furyl)acryloyl]-Phe-Gly-Gly (FAPGG) as substrate, as described by Shalaby et al. [52] with some modifications. Briefly, 10 µL of ACE solution (0.25 unit/mL distilled water) and 10 µL of each hydrolysate sample solution were placed in one well of a 96-well microtiter plate. The microplate was immediately transferred to the microplate scanning spectrophotometer (SpectraMax M5 spectrophotometer, Molecular Devices LLC, USA) thermostated at 37 °C. A sample of 150 µL of substrate solution (0.88 mM FAPGG in 50 mM Tris–HCl, pH 7.5, containing 0.3 M NaCl), preheated at 37 °C, was placed into each well to start the reaction. The absorbance at 340 nm was recorded every 60 s for 60 min. The control consisted of samples containing 10 µL of buffer (50 mM Tris-HCl, pH 7.5, containing 0.3 M NaCl) instead of hydrolysate. Triplicate samples and controls were used in these studies. The ACE activity was determined from the average slope of the absorbance vs. time curve in its linear section, and the ACE inhibition (%) was calculated as reported in Equation (1) below, where pA_inhibitor_ and pA_control_^+^ are the slopes determined with the hydrolysate sample and control samples, respectively.

% inhibition ACE I = [1 − (pA_inhibitor_/pA_control_^+^)]·100 (1)

The concentration–response curves were obtained by assaying various dilutions of the samples and plotting the ACE inhibition percentage as a function of protein concentration. The concentration causing 50% of ACE inhibition (IC_50_), obtained from triplicate samples, was calculated by the mathematical models proposed by Estévez et al. [53].

#### 2.13.3. Anti-Inflammatory Capacity

An anti-inflammatory assay uses the murine monocyte–macrophage cell line RAW 264.7 (ATCC TIB-71). Cells were cultured in Dulbecco’s Modified Eagle Medium (DMEM) (ATCC-30-2002) supplemented with 10% heat-inactivated FBS and antibiotic (solution of 10,000 units of penicillin and 10 mg streptomycin/mL) at 1% and maintained at 37 °C and 5% CO_2_. The anti-inflammatory assay includes a preview determination of cell viability according to the methodology described above which was calculated as % of viability with respect to the control. For each hydrolysate, the highest concentration that does not affect cell viability is selected for the anti-inflammatory assay. The anti-inflammatory capacity of selected concentrations of hydrolysates was evaluated. Exponentially growing RAW 264.7 macrophages were plated in 96-well plates at a density of 2 × 10^5^ cells per well in 100 µL of culture medium, allowing them to adhere overnight. The cells were then stimulated with LPS (100 ng/mL), and the plates were incubated for 30 min in optimal culture conditions. The cells were then treated with positive-control hydrocortisone (100 µM) or samples dissolved in culture medium to the selected concentration for each hydrolysate and incubated at 37 °C, 5% CO_2_, for 24 h. In the plate, the cells were maintained as the LPS control, without treatment, and media control, without LPS or treatment. Cell-free supernatants were collected and used to measure NO, TNF-α, and IL-6. NO-content was determined by the Griess reaction kit (Invitrogen, Thermo Scientific, Waltham, MA, USA), while enzyme-linked immunosorbent assay (ELISA) kits were used for interleukin 6 and tumor necrosis factor (TNF-α) cytokine determination (IL-6 Mouse ELISA Kit and TNF alpha Mouse ELISA Kit; Invitrogen Thermo Fisher, Waltham, MA, USA). The kits were used following the manufacturer’s recommendations. For nitrites, IL-6, and TNF-α, the percentage of inhibition in the production of inflammatory factors by macrophages was calculated for each treatment group using the following Equation (2):

% Inhibition = (1 − [factor]sample/[marker]control LPS) × 100(2)

#### 2.13.4. Anti-Osteoporotic Capacity

An anti-osteoporotic assay explores the influence of compounds on bone growth in vitro. In the present study, the ability of hydrolysate extracts to promote the proliferation and differentiation of osteoblast cells of bone-like matrices on mouse MC3T3-E1 osteoblastic cell subclone 4 (ATCC CRL-2593) was evaluated. The capacity to inhibit the osteoclast proliferation on murine monocyte–macrophage cell line RAW 264.7 (ATCC TIB-71) cells was evaluated in vitro, too. This is expected to give leads about the potential usefulness of hydrolysates as effective anti-osteoporotic agents. The assay includes a proliferative assay of the MC3T3 cell line, a proliferative assay of the RAW 264.7 cell line, and a differentiation assay of the MC3T3 cell line.

- Cell Proliferation

Cell proliferation was measured by an MTT assay. Before treatment with the test compound, cells were seeded in 96-well plates at 5 × 10^3^ cells per well for the MC3T3-E1 cell line and at 2 × 10^4^ cells for each well for the RAW 264.7 cell line. The plates were cultured for 24 h in optimal conditions of growth in alpha essential medium (αMEM) for the MC3T3-E1 cell line and DMEM media for the RAW 264.7 cell line, supplemented with 10% heat-inactivated FBS and 1% of antibiotic solution (10,000 units of penicillin and 10 mg streptomycin/mL) in 5% CO_2_ at 37 °C. To evaluate proliferative efficiency, the media were replaced by a fresh medium containing the test compounds. After treatment with the test compounds for 24 h, an MTT assay was developed. For a comparative standard, the proliferation of cells in the medium without the test compound was defined as 100%.

- Bone Cell Differentiation Test

The MC3T3-E1 cell line was cultured in basal media (BM). The BM was minimum αMEM (Gibcos) supplemented with 10% heat-inactivated FBS and 1% antibiotic solution (10,000 units of penicillin and 10 mg streptomycin/mL). The cells were maintained at 37 °C in a 5% CO_2_ atmosphere. The MC3T3-E1 cells (5 × 10^4^ cells/mL/well) were pre-cultured in BM for 24 h in 24-well plates. The medium was then changed to osteogenic media (OM) with or without the test substances on day 0 and was renewed every 2 days until the day of the end of the experiment. The OM was BM supplemented with 50 μg/mL ascorbic acid, 5 mM β-glycerophosphate, and 10 nM dexamethasone [54,55]. Changes in alkaline phosphatase (ALP) activity, the first bone marker enzyme released during the differentiation process, were quantified because these changes reflect mature osteoblast formation and have been accepted as an early osteoblast activity marker [56]. To determine ALP activity, the cells were washed twice with phosphate buffer prior to lysing the cells with lysis buffer (0.5% Triton X-100 in 50 mM Tris-HCl buffer, 150 mM NaCl, pH 7.4, 400 μL/well) for 30 min at room temperature. The cell lysate was collected separately and centrifuged at 13,000 × *g* for 15 min. The supernatants were stored at −20 °C until the ALP activity was determined. ALP activity was determined by the Alkaline Phosphatase Activity Colorimetric Assay Kit (K412-500, BioVision, San Francisco, CA, USA) and calculated as nmol of p-NP produced/mg protein/min and expressed as % relative ALP activity compared with the control. To normalize the differences in protein content between wells, the protein concentration of each well was determined by the Pierce protein assay (Thermo Scientific, Waltham, MA, USA) [46].

#### 2.13.5. Hepatoprotective Effect against the Accumulation of Fatty Acids in Liver Cells

The assay is performed using the liver cell line HepG2 (ATCC^®^ HB-8065™). Cells were cultured in EMEM (ATCC-30-2003) supplemented with 10% of heat-inactivated FBS, antibiotic (solution of 10,000 units of penicillin and 10 mg streptomycin/mL) at 1% and were maintained at 37 °C and 5% of CO_2_. The hepatoprotective assay includes a preview determination of cell viability according to the methodology described above and was calculated as % of viability with respect to the control. For each hydrolysate, the highest concentration that does not affect cell viability is selected for hepatoprotective assay.

The selected concentrations of hydrolysates were tested on the in vitro hepatoprotective assay. Exponentially growing HepG2 cells were plated in 96-well plates at a density of 5 × 10^3^ cells per well in 100 µL of culture medium, allowing them to adhere overnight. The cells were then stimulated with samples dissolved in a culture medium to the selected concentration for each hydrolysate and incubated at 37 °C, 5% CO_2_, for 24 h. Control wells were maintained, without treatment, only with culture media. Subsequently, the cells were washed twice with PBS (without Ca^2+^ and Mg^2+^) and then treated with culture medium EMEM without FBS supplemented with a mixture of oleic and palmitic fatty acids (proportion 2:1. 1 mM) and 1% BSA. Control without fatty acids is maintained in wells with each treatment, and without treatment, these are incubated with the same volume of culture medium EMEM without FBS and 1% BSA. The plates are incubated again at 37 °C for 24 h, and after this time, the accumulation of lipids in the cells is analyzed with AdipoRed™ Assay Reagent (Lonza), following the manufacturer’s instructions. The results were expressed as an average of the relative percentage of inhibition in fatty acid accumulation with respect to the control without hydrolysates.

### 2.14. Statistical Analysis

All the tests were performed in duplicate or triplicate, and the result is shown as the mean and standard error and/or standard deviation. A statistical analysis was carried out using the *t*-test, considering differences in which a *p* ≤ 0.05 was obtained as statistically significant.

## 3. Results and Discussion

### 3.1. Microbiological Analyses and Evaluation of Lipid Oxidation before and after the Patented Dehydration Process

The extraction material, TFP, appeared as a non-homogeneous powder obtained after a patented mild dehydration treatment performed by Themis S.p.A, starting from the side streams of cooked tuna coming from the canning industry Generale Conserve. The side streams were composed of skin and tuna fillet leftovers that were produced during the process of trimming the tuna fillets after a steam cooking step and before their canning and subsequent sterilization in jars. TFB was collected and immediately stored at −18 °C before being treated by Themis S.p.A, as described above. This treatment yields a lower volume of fish side streams that is more stable over time [28].

To evaluate the impact of this dehydration process, microbiological analyses were carried out (Table 1), and the oxidative states of the pre-and post-dehydration materials (TFB and TFP) were evaluated with the PV test and with the TBARS assay for the primary and secondary oxidation products, respectively.

The chosen analytical strategy for assessing this supply chain for microbiological concerns considered various potential sources of microbiological contaminants that can enter the fish food chain at any stage of the fish’s life cycle. It is also important to underline that all manipulative procedures were carried out outside of sterile procedures, which can increase the possibility of contamination. Naturally, in an industrial setting, it would be simpler to enhance extraction and manufacturing processes by adhering to best practices and risk management to prevent contamination. Microbiological contaminants could originate from the natural environment or be connected to *Salmonella* species and *Enterobacteriaceae*, which are found in the fish’s gut microbiome. Moreover, there are several ways in which these contaminants could enter the supply chain: contaminated facilities, terrestrial environments, or even through contact with operators handling the fish. Contaminants such as *Escherichia coli* are prominent instances of the latter circumstance, representing warning signs of fecal contamination. Therefore, it is essential for food safety that they are absent or barely present (<10 CFU/g), as in this case.

The presence or absence of *Staphylococcus aureus*, a bacterium that can cause food poisoning, is indicated by the coagulase-positive *Staphylococci* value at 37 °C, which was found to be less than 10 CFU/g. *S. aureus* should also be extremely rare or absent in this situation. Furthermore, food safety is also guaranteed by the absence of two organisms that might result in serious illnesses: *Listeria monocytogenes* and *Salmonella* spp.

The presence of sulfite-reducing bacteria is typically considered as a sign that animal products may be contaminated with Clostridial bacteria. Also, in this case, the results were satisfactory (<10 CFU/g).

Histamine is a critical marker of fish quality. It can demonstrate a decrease in quality due to interruptions in the cold chain, which is essential for maintaining freshness and safety during processing. In fact, the fish can experience bacterial overgrowth at temperatures higher than 4 °C, which leads to the conversion of histidine to histamine. Histamine levels in fish that have been properly preserved are typically less than 0.1 mg per 100 g; but, in fish that have been contaminated, histamine levels can reach 20 to 50 mg per 100 g of fish, most frequently due to *Salmonella* spp., *E. coli*, and other bacteria. When consumed, histamine is responsible for mast cell degranulation that mimics an allergic reaction and is comparable to that seen in allergy-mediated histamine response. For this reason, it is very important to keep this parameter monitored and to remain inside the regulated limits for nutraceutical and cosmetic purposes (EU Regulation 2073/2005).

For the TVC, the average data clearly show that the dehydration treatment may have bactericidal effects since a decrease to a logarithm (base 10) is observed in the analyzed samples.

Regarding lipid oxidation, it is considered one of the principal causes of deterioration of muscle-based foods, such as fish, and it leads to the production of rancidity-related taste, which eventually makes the product unfit for human ingestion and leads to a more general loss in nutritional quality [57]. For these reasons, the PV test and the TBARS assay were performed to assess the impact of the patented dehydration process on the fish powder. In fact, because of lipid oxidation, food products’ shelf life is shortened, and food quality deteriorates very fast, causing possible diseases when consumed [11]. The results obtained were reported in Table 2, and they show that no relevant increase in lipid oxidation occurred.

Looking at all the abovementioned results, it is possible to affirm that the dehydrated biomass is stable and safe, and the lipid oxidation is acceptable [58]. For this reason, it can be considered a starting material for extracting protein hydrolysates.

### 3.2. Characterization of Starting Material: pH and Proximate Analysis

TFP was characterized by measuring its pH, which was equal to 5.7 ± 0.3. The proximate analysis was evaluated, obtaining the following results, expressed as %, i.e., g/100 g: residual moisture 5.09 ± 0.33, crude proteins 71.59 ± 1.36, ashes 11.39 ± 1.78, and lipids 11.93 ± 0.70. The proximate composition results are similar to those of the tuna side stream meal reported by Kim et al. [59], with 7% moisture, 68% crude proteins, 12% lipids, and 17% ashes.

Shaviklo reported that if fish protein powders have a protein level higher than 65%, they can be categorized as a fish protein concentrate [9]; so, given the results obtained, and, in particular, the high content of the crude protein fraction, TFP was considered a first-rate starting raw material for the extraction of fish protein hydrolysates, emphasizing the importance of working with concentrated biomass that can be hydrolyzed in order to obtain precious high-value marine peptides.

### 3.3. Yields and Characterization of Tuna Protein Hydrolysates: pH and Proximate Analysis

The obtained yield of EH and NEH were relatively similar, with 23.50% ± 2.35 and 21.70% ± 1.58, respectively. No substantial increase in yield was observed with the addition of the enzyme. This is not unexpected as a large proportion of the proteins in TFB is most likely insoluble after the initial cooking process. This observation was also supported by previous experiments, where the hydrolysis process using enzymes resulted in a large residual solid phase still containing high amounts of protein (around 65%).

The pH of both the hydrolysates, EH and NEH, which were demonstrated to be highly soluble in water, was measured, giving results of 6.23 ± 0.02 and 6.27 ± 0.01, respectively.

As for the characterization of EH and NEH, their compositions in terms of proximate analysis are reported below in Table 3.

There are no big differences in terms of proximate composition; in particular, the residual moisture is strongly dependent on the conditions applied during the spray-drying step, which were the same in both cases, and the results are comparable with the ones reported in the review by Chalamaiah et al. [60] where they found that the residual moisture of different fish protein hydrolysates is usually below 10%. The main finding is the crude protein content which is similar for the two protein hydrolysates, and it is higher than the ones obtained by Dana et al. from tuna (72% for tuna dark-meat hydrolysates and 70% for tuna white-meat hydrolysates) [61]. Ash content (9–10%) is considered a good value if compared to the previous values obtained by the authors adding NaOH to reach the optimum pH of 9.0 for the enzyme activity. In this case, ash content ranged around 30%, which may not be considered a feasible solution and, for this reason, was deliberately avoided by the authors.

The lipids were found in lower percentages, but higher in the case of EAE, and the results were similar to those obtained by Vázquez et al. for canning tuna waste protein hydrolysates in the range of 2–5 kDa, which were around 4% [62].

### 3.4. Color Analysis of Tuna Protein Hydrolysates

**Figure 2:**
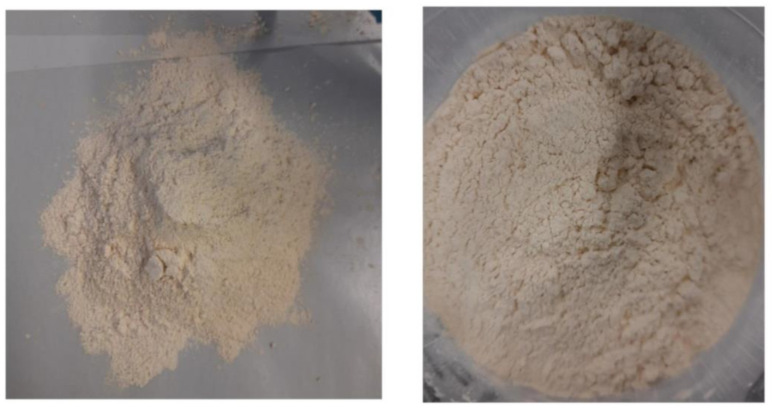
Tuna protein hydrolysates: EH (Enzymatic Hydrolysates) and NEH (Non-Enzymatic Hydrolysates).

Figure 2 reports the two types of tuna protein hydrolysates (EH and NEH), and Table 4 shows their color profiles.

Compared to tuna protein hydrolysates from viscera, which has a brownish-yellow coloration (L* = 62.46, a* = 1.44 and b* = 23.05), as reported by Klomklao et al. [63], EH and NEH have a lighter and yellow coloration. Darker and reddish colors were registered by Sathivel et al. [64] for salmon protein hydrolysates, probably due to the retention of lipids (around 24%) and apolar pigments in the products. The lighter and whiter color of these hydrolyzed proteins provides an important feature as potential raw materials of formulable products in both the nutraceutical and cosmetic sectors.

### 3.5. Amino Acid Composition of Tuna Protein Hydrolysates

**Figure 3:**
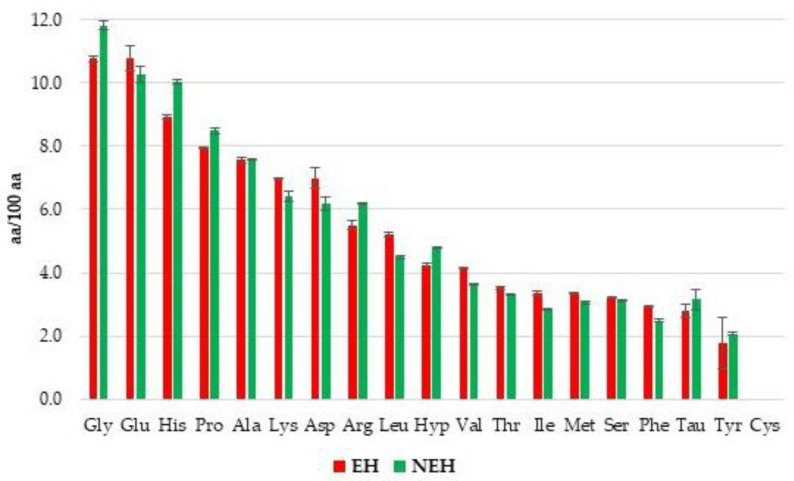
The amino acid profiles of EH (Enzymatic Hydrolysates) and NEH (Non-Enzymatic Hydrolysates).

The compositions of the amino acid profile of EH and NEH were investigated by HPLC, and the results are reported in Figure 3, expressed in %.

The two profiles follow the same trend, meaning that adding an exogenous enzyme did not affect the amino acid compositions of the two protein hydrolysates. They both are characterized by a high content of Glycine (11–12%), Glutamic Acid (10–11%), Histidine (9–10%), Proline and Alanine (both about 8%), Lysine and Aspartic Acid (6–7%), Arginine (around 6%), and Leucine and Hydroxy Proline (both about 5%).

The analysis is particularly interesting since the role of the amino acid composition in dietary protein hydrolysate is noteworthy in multiple physiological activities [65].

Due to the presence of amino acids that have hydrophobic side chains, such as Glycine and Alanine in high concentrations, but also Proline, Leucine, Valine, Isoleucine, Methionine, and Phenylalanine even if in lower concentrations, the bioavailability is strongly improved thanks to the interactions with hydrophobic targets, like the cell membrane [66].

As for the possibility of using these protein hydrolysates in food supplements, the essential amino acids (Histidine, Isoleucine, Leucine, Lysine, Methionine, Phenylalanine, Threonine and Valine) together constitute 37% of the total amino acid composition of both EH and NEH, and in particular, between them, the most important are the branched-chain amino acids (BCAAs) (Isoleucine, Leucine, and Valine) [67] that made up for 12% of the total amino acid composition. It must be underlined that in this case, the amino acid Tryptophane was not quantified, therefore, given that it is an essential amino acid, the hydrolysates can be considered suitable for use as ingredients of a protein supplement, since usually the percentage of essential amino acids in protein supplements is about 40%. Moreover, a good protein supplement generally contains about 15% of BCAAs [68], so the results can be considered satisfactory [69].

### 3.6. FTIR Analysis of Tuna Protein Hydrolysates

**Figure 4:**
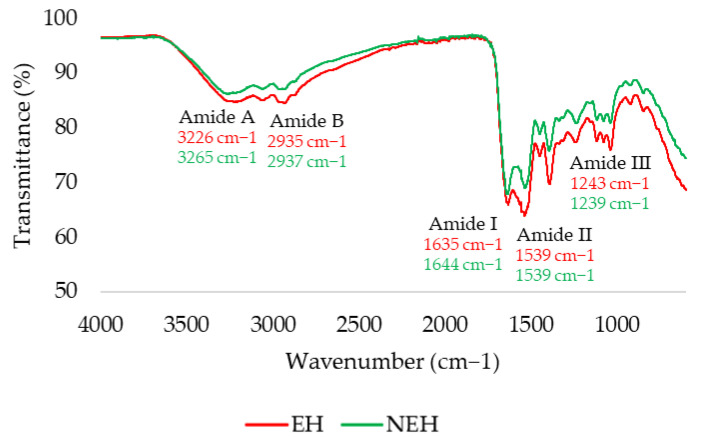
Spectra of EH (Enzymatic Hydrolysates) and NEH (Non-Enzymatic Hydrolysates).

The ATR-FTIR spectra, reported in Figure 4, were registered to reveal structural information of both the side chains of amino acids and the protein hydrolysates’ backbone [70]. In particular, the two spectra are characterized by the main principal peaks that are considered typical of Amides A, B, I, II, and III.

As expected, the two spectra do not show evident differences, so it can be deduced that both samples had the same functional groups, and they present little variations in peak amplitudes [71]. Amide A is represented as a large band at 3226/3265 cm^−1^, and it is characteristic of intermolecular H-bonds associated with N-H stretching; while Amide B is associated with both ^+^NH3 and the asymmetric stretching vibrations of =CH [72], with quite low intensity peaks at 2935/2937 cm^−1^. Mainly connected to the secondary structure (α-helix and β-sheet), the Amide I peak resulted from the CO stretching vibration of the peptide bonds [73], and it is found at 1635/1644 cm^−1^. The reaction of hydrolysis is catalyzed by a proteolytic enzyme that produces COO^−^ terminals and ^+^NH3 terminals, which changes the protein’s or peptide’s primary and secondary structures, respectively. For evaluating the degree of hydrolysis, some of the most accurate markers were the bands of Amide II, found at 1539 cm^−1^ for both the extracts, which represent the ^+^NH3 and the bands at around 1400 cm^−1^, in particular at 1395/1398 cm^−1^ corresponding to COO^−^ [74]. The peaks between 1300 and 1200 cm^−1^, in particular found at 1243/1239 cm^−1^, are considered the ones of Amide III and are mostly linked to vibrations of CN stretching and NH bending (about 30% each), CC stretching (about 20%), and CH bending (about 10%) [75]. In addition to these principal peaks, it is possible to observe a band at 1042 cm^−1^ for both the hydrolysates that is considered as the result of CO, CC, and CN stretching [76].

### 3.7. Molecular Weight Distribution by Size Exclusion Chromatography of Tuna Protein Hydrolysates

SEC was applied to determine the average molecular weight of the hydrolysates, giving 2192 Da ± 15.6 for NEH and 1258 Da ± 7.1 for EH. The relative size distributions of the two protein hydrolysates are summarized in Table 5.

EH presents a lower average molecular weight as expected, compared to NEH. EH and NEH have an average molecular weight below 3 kDa, so they can be classified as “extensively hydrolyzed peptides” [77]. A distribution of molecular weights below 1500 Daltons makes the EH suitable for allergy sufferers since peptides below this threshold are not considered allergenic because they do not contain antigenic epitopes effective for activating immune cells [78,79]. So, hydrolysis reduces the ability of proteins to induce allergenicity, and this is particularly useful for the food industry and the development of hypoallergenic protein supplements, where it is important to minimize the risk of allergic reactions in consumers.

### 3.8. Determination of Antioxidant Capacity of Tuna Protein Hydrolysates

Tuna protein hydrolysates, produced by hydrolysis of tuna side streams, are considered a possible nutraceutical ingredient in the food industry thanks to their texturizing, whipping, and gelling qualities; moreover, they are a possible source of antianemia chemicals, antioxidants (such as peptides with anticancer activity), and ingredients for microbial growth media [80].

Studies on antioxidant peptides have been reported, showing that antioxidant peptides were commonly found to have relatively low molecular weight, simple structure, high activity, easy absorption, better stability in different situations, and no harmful immune reactions [81,82]. Four methods were used to determine antioxidant activity: FRAP, TEAC, and ORAC for determining radical-scavenging activity and the CAA assay for evaluating cell-based antioxidant response. The results are reported independently for each methodology below.

#### 3.8.1. Ferric Reducing Antioxidant Power Assay

It has been demonstrated that protein hydrolysates possess antioxidant properties that prevent lipids and/or fatty acids from peroxidizing [83]. The FRAP test was used to examine the antioxidant activity, and the results were reported as ferrous equivalents (mM) using a calibration curve obtained with the kit’s ferrous ammonium sulfate standard solutions. By employing a redox colorimetric reaction, the test quantifies the compound’s reducing power capacity to convert ferric iron (III) to ferrous iron (II) [45]. A range of concentrations, from 25 to 100 mg/mL, were taken into consideration, which corresponded to ferrous equivalent findings of 0.31 to 1.05 mM and 0.30 to 0.94 mM for EH and NEH, respectively, which were higher than the values obtained by Grasso et al. while evaluating FRAP of hydrolyzed gelatin/collagen peptides [84].

#### 3.8.2. Radical-Scavenging Activity: Trolox Equivalent Antioxidant Capacity and Oxygen Radical Antioxidant Capacity

**Figure 5:**
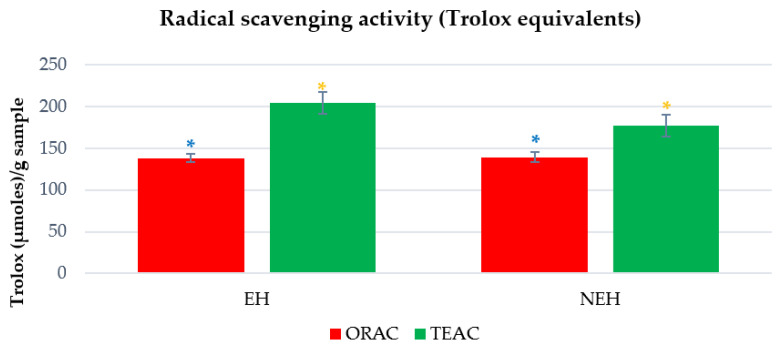
Determination of TEAC (Trolox Equivalent Antioxidant Capacity) and ORAC (Oxygen Radical Absorbance Capacity) for EH (Enzymatic Hydrolysates) and NEH (Non-Enzymatic Hydrolysates). (* Statistics between tests presented with different colors; same colors indicate that there is no significant difference, *p* ≤ 0.05).

The TEAC and ORAC results are expressed compared to the water-soluble vitamin E analog Trolox (in Trolox equivalents) (Figure 5) and as the IC_50_ concentration (Table 6) of a sample that caused a 50% decrease in the initial radical concentration. All the tests are performed in triplicate, and the results are shown as the mean value ± standard error.

The size of the peptides obtained after enzymatic hydrolysis determines many of the functionalities of the hydrolysates. The antioxidant activity of peptides is affected by their structural characteristics, especially their molecular masses, amino acid composition, and peptide sequence. It has been reported previously that peptides consisting of 2–13 amino acids with molecular mass ranging from 268 to 1450 Da are likely to show great antioxidant capacity [85,86]. Samples with a lower molecular weight have a better ability to donate electrons and hydrogen that will react with free radicals. Peptides with a lower molecular weight have a more significant amount of hydrogen to react and stabilize free radicals by stopping free radical chain reactions [87,88].

#### 3.8.3. Cellular Antioxidant Activity Assay

**Figure 6:**
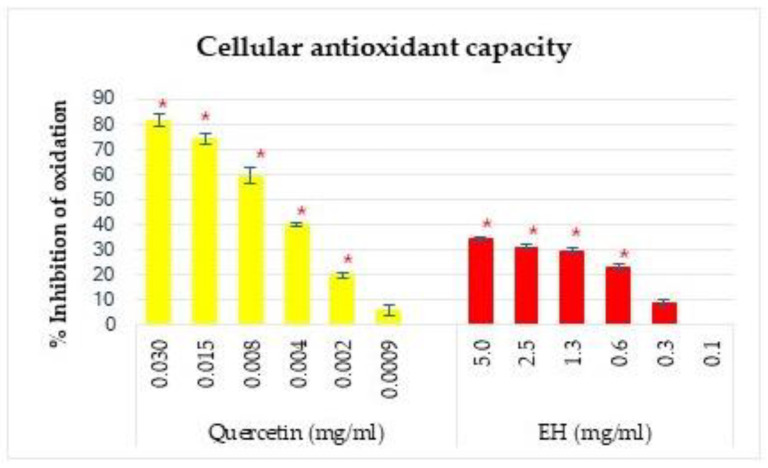
Cellular Antioxidant Activity (CAA) of the tuna protein hydrolysates EH (Enzymatic Hydrolysates); * significant differences (*p* < 0.05).

A CAA assay is an antioxidant cell-based assay. Biochemical assays are target-based assays, while cell-based assays are physiology-based assays. Functionally, biochemical assays analyze the activity of biomolecules quantitatively and qualitatively, while cell-based assays allow the detection of the response of biological organisms to a particular substance or biomolecule [89]. The results of CAA are expressed as percentage inhibition of oxidation as a function of concentration (Figure 6). It is impossible to express the IC_50_ reliably since, in most cases, the highest concentration that allows cell viability does not reach 50% inhibition and would, therefore, be an estimated value.

The NEH sample did not show CAA activity. This could be explained based on the molecular weight of the sample, as hydrolysates with molecular weights below 3 kDa show CAA activity. Molecular weight is a determinant factor for cell absorption, but other factors, such as solubility or polarity of the wide variety of compounds present in hydrolysates, give each of them unique permeability, allowing for bioactivity [90]. For the EH sample, the observed effect is similar, but the maximum observed capacity is 35% inhibition at concentrations above 0.6 mg/mL. The results are in discrepancy with radical-scavenging activity; this could be explained by differences in cell permeability. The chemical structure of the compounds present in the hydrolysates could determine that they can be broken down by certain hydrolytic enzymes in the HepG_TR_EFP2 cell membrane when they enter the cells, thus affecting their antioxidant capacities [91]. An improvement in cell-based models over chemical models is the assessment of the available antioxidant activity of compounds. The observed effects on the biological activities of an extract or hydrolysate in a cellular system, in fact, are a consequence of the compound portions absorbed by the cells [92,93,94].

Despite the discrepancies in results, the information provided by the tests can be applied differently. The CAA assay is more representative of biological systems, but radical-scavenging assays provide information on the antioxidant capacity of the directly applied compound. This is useful if their purpose is not to provide this capacity within a biological system, e.g., as an oxidation prevention compound, which allows the prolongation of a food’s shelf life.

### 3.9. Additional Biological Tests of Tuna Protein Hydrolysates

#### 3.9.1. ACE-Inhibitory Activity (Antihypertensive Capacity)

**Figure 7:**
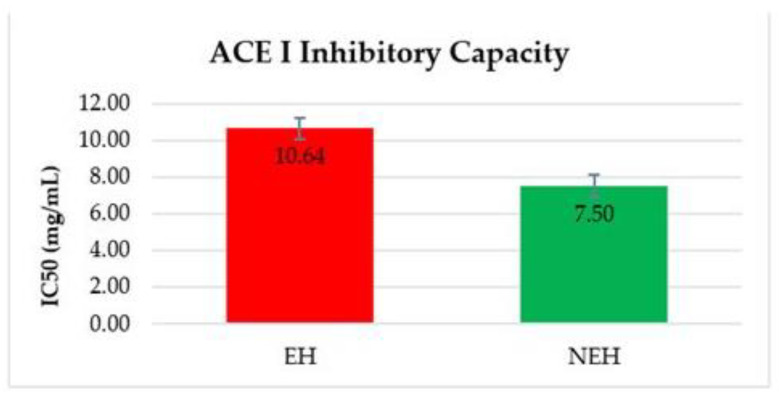
Antihypertensive activity of EH (Enzymatic Hydrolysates) and NEH (Non-Enzymatic Hydrolysates).

The samples were analyzed using the described methodology in triplicate, and the values were shown as averages, where the bars indicate the error (Figure 7). The results are expressed as IC_50_. A comparison was made with a known compound, to get an idea of the antihypertensive capacity of the extracts studied; the test was also performed with a compound currently used as a medical treatment for hypertension, i.e., Captopril. This compound shows an IC_50_ for ACE I at 0.013 ng/mL.

Both EH and NEH showed a promising antihypertensive activity equal to IC_50_ 10.64 and 7.50 mg/mL, respectively. Therefore, the NEH sample required a lower concentration to inhibit the same enzyme activity. This result is in line with the findings of most of the studies, which reflect that molecular size significantly impacts the affinity between peptides and ACE because large peptides cannot pass through the narrow binding channel of ACE. And this is directly related to the degree of hydrolysis achieved in the process and to the ability of enzymes to generate peptides with high affinity for ACE-binding sites [95].

#### 3.9.2. Anti-Inflammatory Capacity

**Figure 8:**
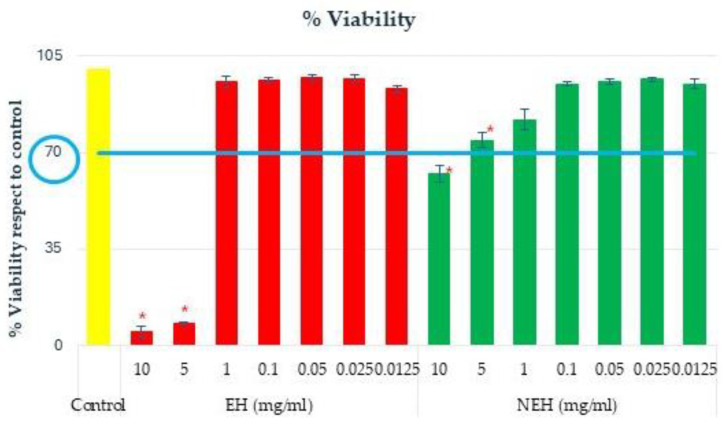
Cell viability in response to sample concentration (*) significant differences (*p* < 0.05) of the protein hydrolysates EH (Enzymatic Hydrolysates) and NEH (Non-Enzymatic Hydrolysates). If cell viability is < 70% (under blue line), the sample is considered to have cytotoxic potential.

To perform the anti-inflammatory capacity assay, it is necessary to determine the cytotoxicity of the samples on RAW 264.7 cells. Cell viability was determined by the MTT assay (Figure 8). If cell viability is <70%, the sample is considered to have cytotoxic potential [96]. At the three highest concentrations of EH and NEH (10, 2, and 1 mg/mL), a reduction in viability below 70% is observed.

**Figure 9:**
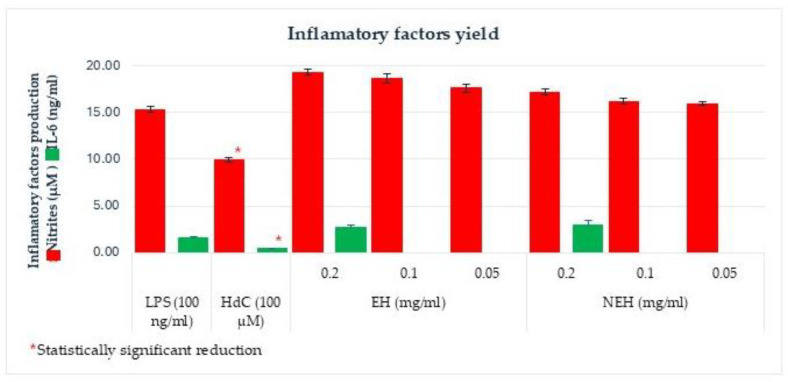
Inflammatory factor production of the hydrolysates from tuna byproduct EH (Enzymatic Hydrolysates) and NEH (Non-Enzymatic Hydrolysates) in response to sample concentration (* significant differences (*p* < 0.05) with respect to LPS).

For the anti-inflammatory assay, the maximum concentration that does not significantly reduce cell viability and the two concentrations below were selected (0.2, 0.1, and 0.05 mg/mL). Inflammation was induced with LPS, and the effect of the samples on the inflamed cells was evaluated. For this purpose, the levels of nitrites and IL-6 produced by the cells were determined. The sample was considered to have an anti-inflammatory effect when the production of inflammatory factors was statistically significantly lower (*t*-test *p* < 0.05) with respect to treatment with LPS (Figure 9). Hydrocortisone (HdC) was used as a positive anti-inflammatory control.

The assayed hydrolysates did not show anti-inflammatory capacity since they did not significantly inhibit the production of the inflammatory factors studied. These results could be related to the initial dehydration treatment of the raw material; in fact, heat denaturation is one of the main causes that could alter the functional capacity of proteins.

#### 3.9.3. Anti-Osteoporotic Capacity

In the present study, the ability of hydrolysate extracts to promote the proliferation and differentiation of osteoblast cells of bone-like matrix on mouse MC3T3-E1 osteoblastic cell subclone 4 (ATCC CRL-2593) was evaluated. In addition, the capacity to inhibit osteoclast proliferation on murine monocyte–macrophage cell line RAW 264.7 (ATCC TIB-71) cells was evaluated in vitro, too.

- Cell Proliferation

**Figure 10:**
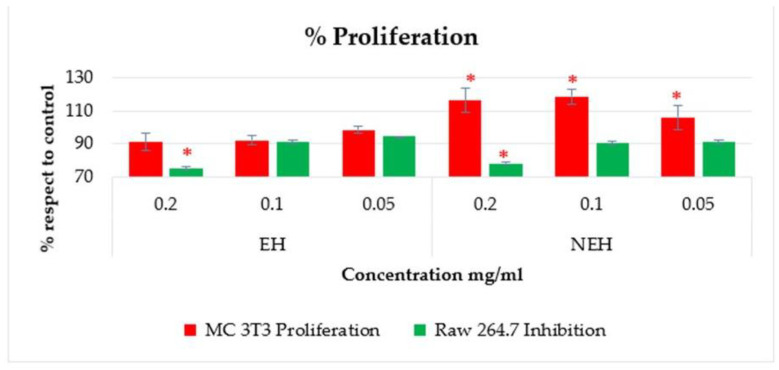
Osteoblast proliferation and osteoclast inhibition of EH (Enzymatic Hydrolysates) and NEH (Non-Enzymatic Hydrolysates). (*) significant differences (*p* < 0.05).

In the assay, the proliferation capacity of the osteoblast was determined in the presence of the hydrolysates. In all cases, optimal growth is observed at concentrations below 0.2 mg/mL, similar to that of the control. Values greater than 100% imply cell proliferation since the viability is higher than that which occurs under optimal growth conditions, but no increase in cell proliferation is observed (Figure 10).

Osteoclast inhibition was also determined by the sample at the concentration selected for the anti-osteoporotic capacity study (the highest concentration that does not affect the viability of MC 3T3). The results show that at the concentrations used, the viability of osteoblasts is similar to that of the control, increasing only for the NEH sample. However, the viability of osteoclasts is significantly reduced in samples with 0.2 mg/mL compared to the control without sample.

- Bone Cell Differentiation Test

**Figure 11:**
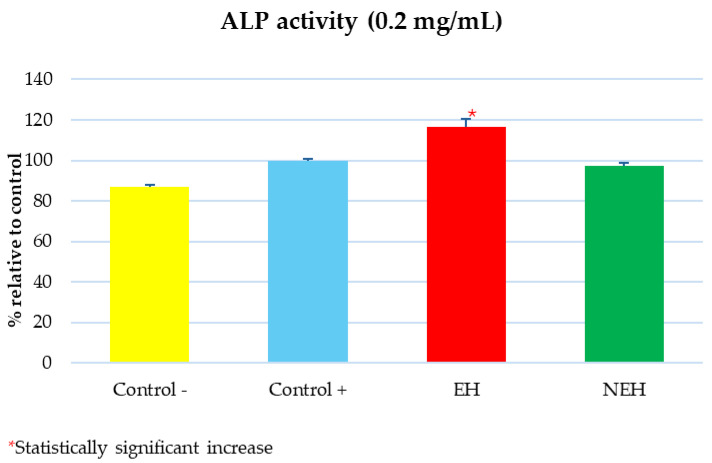
Comparison of osteoblast differentiation activity of EH (Enzymatic Hydrolysates) and NEH (Non-Enzymatic Hydrolysates); * significant difference (*p* < 0.05).

Changes in alkaline phosphatase (ALP) activity, the first bone marker enzyme released during the differentiation process, were quantified because these changes reflect mature osteoblast formation and have been accepted as an early osteoblast activity marker [56]. The results are shown as an average of the relative percentage of ALP activity. Only the EH sample increased the % ALP statistically significantly with respect to the positive control. The ALP concentration in the negative control was, as expected, significantly lower than in the positive control, as shown in Figure 11. No anti-osteoporotic capacity is observed in the other tested NEH samples.

#### 3.9.4. Hepatoprotective Effect against Accumulation of Fatty Acids in Liver Cells

**Figure 12:**
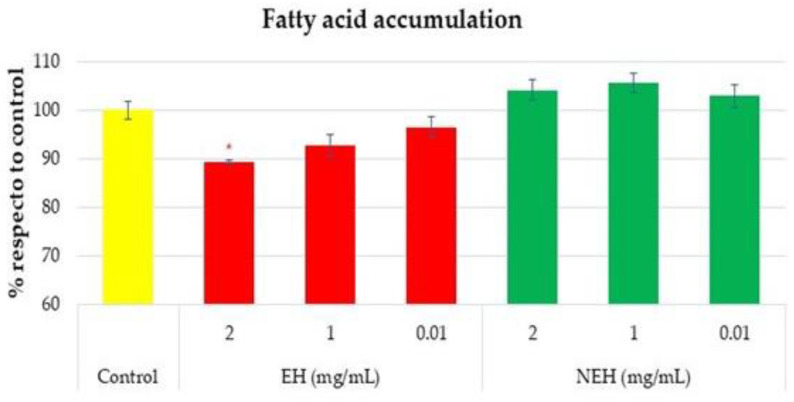
Comparison of fatty acid accumulation, * significant difference (*p* < 0.05) of EH (Enzymatic Hydrolysates) and NEH (Non-Enzymatic Hydrolysates).

A hepatoprotective assay includes a preview determination of cell viability in the presence of variable concentrations of the sample to be tested, as previously reported in Figure 8. Hepatoprotective assays were performed in triplicate with sample concentrations of 2, 1, and 0.01 mg/mL. The results are shown as an average of the relative percentage of inhibition in fatty acid accumulation with respect to the control without hydrolysates (Figure 12).

The protective effect of the accumulation of fatty acids in hepatic cells is based on the reduction in fatty acid content in the presence of tested hydrolysates. The result reflects a reduction in cells treated with low-molecular-weight hydrolysates (EH), which could be related to the capacity to cross the cell membrane. This result agrees with the study by Huang, who reported the efficacy of tuna dark-muscle hydrolysate in lipid regulation [97].

## 4. Conclusions

This work aimed to answer the urgent call for a blue circular economy in view of a “zero waste” approach by exploiting the side streams generated by the tuna canning industry. The authors dealt with a particular starting material that is an unsorted, mixed matrix composed of leftover cooked tuna fillet and trimming residues, after being dehydrated with a mild technology, currently under patent. This treatment provides significant benefits in handling biomass logistics by consistently reducing volume and weight, allowing it to work with a concentrated starting material. Additionally, it has been shown that this treatment is essential for extending the storage of perishable biomass. Microbiological analyses and lipid oxidation tests have shown reassuring results.

This work proposed two simple extraction protocols with and without the addition of an Alcalase enzyme. This study revealed that the addition of enzyme did not affect the hydrolysate yield but influenced the average molecular weight (MW) of the hydrolysates. In both cases, very extensively hydrolyzed peptides that are highly soluble in water with an average MW below 3 kDa were obtained. In the case of the hydrolysate produced using enzymes, the average MW was under 1.5 kDa, which can be considered hypoallergenic. Both the two protocols allowed yields higher than 20 g of hydrolysates from 100 g of starting material; moreover, the proximate analysis of the aforementioned was evaluated, consisting of a very high percentage of proteins, i.e., around 80%, and the analysis of the amino acid profile revealed that about 40% of the total amino acids are composed of essential ones, with 12% of BCAAs, so that both hydrolysates can be considered in a formulation of food and feed supplement.

Given the promising results and considering the amino acid profiles and the molecular weight distributions, several biological tests were performed to assess a range of potential bioactivities, including antioxidant, ACE-inhibiting, anti-osteoporotic, and hepatoprotective effects against the accumulation of fatty acids in liver cells. Particular attention was paid to antioxidant activity, as it is fundamental for food applications and other industrial sectors (e.g., biomaterials for edible packaging/coating). The tests carried out to determine antioxidant capacity in vitro revealed that the results on cellular models are different from TEAC and ORAC, possibly due to cell permeability. Nevertheless, the cellular model EH shows the most promising results. Nevertheless, the cellular model EH shows the most promising results due to the fact that the antioxidant activity of peptides is affected by their structural characteristics, especially by their molecular masses.

The determination of ACE-inhibitory activity selected NEH as the best candidate for this purpose, even though EH also showed antihypertensive capacity. Differently, none resulted having an anti-inflammatory capacity. Both the bioactives (EH and NEH) showed anti-osteoporotic capacity, and EH also revealed a protective effect from the accumulation of fatty acids in hepatic cells (at a concentration equal to or lower than 2 mg/mL).

In general, EH resulted in the best compromise for nutraceutical purposes.

## Figures and Tables

**Table 1:** Microbiological analyses on starting material before (TFB) and after (TFP) the dehydration process.

Microbiological Analyses ^1^	TFB	TFP
Enterobacteriaceae (CFU/g)	<10	<10
Escherichia coli β-gluc, +(CFU/g)	<10	<10
Coliforms (CFU/g)	<10	<10
Coagulase + Staphylococci at 37 °C (CFU/g)	<10	<10
Salmonella spp. (in 25 g)	Absent	Absent
Listeria monocytogenes (in 25 g)	Absent	Absent
Sulfite reducing Clostridia and spores (CFU/g)	<10	<10
Histamine (mg/kg)	<5.0	<5.0
Total Viable Count (CFU/g)	43,750	4650

^1^ Results are reported as average value (n = 3).

**Table 2:** Status of lipid oxidation of the starting material before (TFB) and after (TFP) the dehydration process with Peroxide Value (PV) and Thiobarbituric Acid Reactive Substances (TBARS).

	TFB	TFP
PV (meq O_2_/kg) ^1^	1.45 ± 0.19	1.63 ± 0.03
TBARS (mmol MDA/kg) ^1^	9.24 ± 1.23	11.37 ± 0.22

^1^ Results are reported as average ± standard error.

**Table 3:** Proximate analysis of EH (Enzymatic Hydrolysates) and NEH (Non-Enzymatic Hydrolysates).

	EH (g/100 g) ^1^	NEH (g/100 g) ^1^
Residual moisture	5.61 ± 0.22	5.35 ± 0.15
Crude proteins	80.88 ± 0.06	83.17 ± 1.84
Ashes	8.76 ± 0.09	10.05 ± 0.05
Lipids	4.76 ± 1.61	1.43 ± 0.14

^1^ Results are expressed as mean ± standard error (n = 2).

**Table 4:** CIELab color space analysis of EH (Enzymatic Hydrolysates) and NEH (Non-Enzymatic Hydrolysates).

	EH ^1^	NEH ^1^
L*	89.5640 ± 0.14	88.1375 ± 0.76
a*	−0.5417 ± 0.01	−0.5550 ± 0.01
b*	13.0496 ± 0.07	11.8521 ± 0.18

^1^ Results are expressed as mean ± standard error (n = 2).

**Table 5:** Relative size distributions of EH (Enzymatic Hydrolysates) and NEH (Non-Enzymatic Hydrolysates).

Molecular Weight Ranges (Da)	EH (Relative Amount %) ^1^	NEH (Relative Amount %) ^1^
**2000+**	17.2 ± 0.4	34.4 ± 0.3
**1000–2000**	34.8 ± 0.5	29.7 ± 0.1
**500–1000**	37.8 ± 0.6	25.5 ± 0.2
**0–500**	10.3 ± 0.7	10.5 ± 0.1

^1^ Results are expressed as mean value ± standard deviation (n = 2).

**Table 6:** Radical-scavenging capacity results for TEAC (Trolox Equivalent Antioxidant Capacity) and ORAC (Oxygen Radical Absorbance Capacity) expressed as IC_50_ and Trolox equivalents of EH (Enzymatic Hydrolysates) and NEH (Non-Enzymatic Hydrolysates).

**IC_50_ (mg/mL)**	**TEAC ^1^**	**ORAC ^1^**
**EH**	0.80 ± 0.077	0.49 ± 0.014
**NEH**	0.93 ± 0.097	0.53 ± 0.012
**Trolox**	0.04 ± 0.002	0.02 ± 0.001
**Trolox eq (µmol/g sample)**	**TEAC** ^1^	**ORAC** ^1^
**EH**	204.17 ± 12.754	137.82 ± 4.680
**NEH**	176.88 ± 12.641	138.98 ± 6.271

^1^ Results are expressed as mean value ± standard error.

## Data Availability

Data are contained within this article.

## Abbreviations

AAPH	2,2,-azobis(2-methylpropionamidine) dihydrochloride
ABTS	2,2′-azino-bis (3-ethylbenzothiazoline-6-sulfonic acid)
ALP	Alkaline phosphatase
αMEM	Alpha essential medium
ATR-FTIR	Attenuated Total Reflectance –Fourier-Transform Infrared Spectroscopy
BCAAs	Branched-chain amino acids
BM	Basal media
BSA	Bovine serum albumin
CAA	Cellular Antioxidant Activity
DCFH-DA	2′,7′-dichlorofluorescein diacetate
DMEM	Dulbecco’s Modified Eagle Medium
DMSO	Dimethylsulfoxide
EAE	Enzymatic-Assisted extraction
EH	Enzymatic Hydrolysates
ELISA	Enzyme-linked immunosorbent assay
EMEM	Eagle’s Minimal Essential Medium
FAO	Food and Agriculture Organization
FAPGG	N-[3-(2-Furyl)acryloyl]-Phe-Gly-Gly
FBS	Fetal Bovine Serum
FRAP	Ferric Reducing Antioxidant Power
HBSS	Hanks’ Balanced Salt Solution
HEPES	4-(2-Hydroxyethyl)piperazine-1-ethanesulfonic acid
LPS	Lipopolysaccharide
MTT	3-(4,5-dimethylthiazol-2-yl)-2,5-diphenyltetrazolium bromide
MW	Molecular weight
NEH	Non-Enzymatic Hydrolysates
OM	Osteogenic media
ORAC	Oxygen Radical Absorbance Capacity
PBS	Phosphate Buffer Solution
PDA	Photodiode array
p-NP	p-nitrophenol
p-NPP	p-nitrophenyl phosphate
PV	Peroxide Value
SEC	Size Exclusion Chromatography
TBARS	Thiobarbituric Acid Reactive Substances
TEAC	Trolox Equivalent Antioxidant Capacity
TFB	tuna fish biomass
TFP	tuna fish powder
TMP	1,1,3,3-tetramethoxypropane
TVC	Total Viable Count
